# The Early Diagnosis of Pulmonary Tuberculosis

**Published:** 1913-02

**Authors:** Burt R. Shurly

**Affiliations:** Detroit


### The Early Diagnosis of Pulmonary Tuberculosis

By DR. BURT R. SHURLY,
Detroit.

UNTIL a specific is obtained the life of the tuberculous individual depends on early diagnosis and early treatment. A vast number of those infected with the pulmonary form pass through a so-called pretubercular or presymptomatic or latent period. Although we have no tuberculosis without the tubercle bacilli, yet if we postpone our most strenuous efforts until the microscope reveals the lesions the fight is often beyond us. The early forms of latent unrecognized tuberculosis demand more of our attention.

If we hope to accomplish the work of extermination of this dread disease we must constitute each physician an educational unit. He must bear the burden of this great crusade. No specialist can rob him of his responsibilities of guarding the predisposition to tuberculosis of the growing child. It is within his realm to detect the first signs of infection and upon this knowledge and acute observation will depend early diagnosis, the life of the individual, and the prophylaxis of the race.

It may be of interest to review some of the methods of examination that aid in the early detection of pulmonary tuberculosis and to outline some of the fundamental principles that are in vogue at the present time.

There is frequently a prolonged prodromal period of impaired general condition that is marked by a well observed disturbance of the usual ratio between the body weight in pounds and the height in feet, associated with a progressive loss in weight. The external chest conditions, such as conformation, limited expansion or capacity, may indicate the improper use of the pulmonary cells. The character of the pulse may give us additional evidence, such as acceleration with a relative decrease of arterial pressure, lymphatism with decidedly pathologic tonsils, adenoids, and cervical glands, frequently exist. Will power may replace automatic power. Subjective symptoms become prominent, such as general malaise, failing digestion, weakness in the knees, cough and slightly increased or hurried respiration.

This train of symptoms without careful examination, is often taken to mean, coryza, bronchitis, grippe, pleurisy, chronic pneumonia, neurasthenia, malaria, anaemia, or indigestion. When these conditions do not respond readily to treatment they should be looked upon with suspicion and investigated thoroughly.

An early diagnosis of tuberculosis can only be made and confirmed by a careful investigation of every abnormal detail connected with the past and present life history of the patient. General rules of procedure may be followed that afford some suitable classified method of investigation. These include the clinical history, physical examination, and the laboratory. A good record blank is important. One that is practible for office use should not include unnecessary details. The family history is particularly important from the view point of opportunity for infection as well as heredity. Later children are more susceptible to the disease, also those who follow the physical type of the tuberculous parent or his relatives. Heredity is frequently given as a predisposing cause when a case results from direct house or fly infection. Early signs should be sought, especially after typhoid fever, broncho-pneumonia, empyema, pleuritis, influenza, whooping cough, measles or pregnancy with delayed recovery.

In the history it is important to elicit the facts and details in regard to the cardinal symptoms of onsetting pulmonary tuberculosis. Many patients who will deny a cough will admit a clearing of the throat, throat trouble, or catarrh, which, on analysis will quickly assume the form of a dry, hacking, persistent cough of early invasion, the degree of which is often in direct relation to the location of the lesion, although some cases pass on to the moderately advanced stage without the development of cough.

An evening rise of temperature observed for at least three nights with subnormal temperature in the morning is of great importance. This is often attended by languor, malaise or fatigue of the late afternoon and interpreted by the patient as as "the tired feeling" of the patent medicine advertisement. Loss of weight is an important sign that is often unobserved by the patient unless interpreted in terms of loose clothing.

Chloroanemia or secondary anemia may be one of the prominent conditions of the presymptomatic stage. It is extremely marked in some cases. The diminution of the haemoglobin may be out of all proportion to the loss in red cells. Henocque characterized tubercular chloroanemias by a diminished supply of oxyhaemoglobin.

Incipient phthisis usually shows the blood picture of a slight leucocytosis, a slight reduction of red blood corpuscles and a moderate fall in haemoglobin. Gastro-intestinal symptoms are often among the first to follow infection with tubercle bacilli. Anorexia may be exhibited as a gastric unrest. No food is cooked or selected properly for the patient. Light eating and fastidiousness as to dishes may be supplanted under proper treatment with a ravenous appetite and sturdy digestion.

Pain on pressure over the apices or pleuritic pain may be the earliest subjective manifestations of tubercular invasion. No two symptoms develop phthisiophobia or actually alarm a patient in a greater degree than haemoptysis and night or sleep sweats. These are often the first flat argument to the patient, his friends, and too often to the physician that he is truly confronted with a genuine case of pulmonary tuberculosis. Early invasion with bronchial haemorrhage is practically never fatal. As an initial symptom, sleep sweats are rare. They usually mark the presence of well marked physical signs of softening or cavity formation. Hoarseness of persistent type calls for a careful pharyngeal and pulmonary examination.

The committee of education of the American Medical Association has realized that too much special surgery was taught in our medical colleges and the curriculum has been changed to the great advantage of the student. Physical examination and diagnosis are now taught in sophomore, junior, and senior years and time of actual demonstrations in this essential and fundamental branch of internal medicine has been given the importance it deserves. More errors in the diagnosis of pulmonary disease occur from careless examination than from lack of knowledge or ability of the practitioner.

There are certain general rules of physical examination that must be observed. It is unsafe to render an opinion on pulmonary disease until the patient has been stripped to the waist and anterior, posterior and lateral areas, have been carefully examined by the various methods of inspection, palpation, percussion, and auscultation. It should be kept in mind that the earliest physical signs are generally manifest in the posterior right supra-clavicular region. Inspection may show lagging of an affected area on deep inspiration. The acromial end of the clavicle may be lower on the pathologic side. Retraction or depression may be apparent. Inspection will reveal characteristics of size and shape of the chest, conditions of symmetry and comparative mobility, respiratory movements including the prominence or retraction of the intercostal spaces and peculiarities of motion in the diaphragm. Rate and character of respiration. Unilateral wasting in the supra-scapular region.

Palpation gives us information in regard to enlarged cervical axilliary and epitrochlear glands. Tender points. Lagging, palpatory percussion known as finger resistance is often a valuable method of differentiation. Tactile fremitus, friction and rales may be determined. Increased fremitus may be obtained in the early stages.

Percussion, light or heavy will determine the quality and pitch of the note and work out the apical outline. Dullness is of no special value in differentiation, but hyperresonance is one of the most constant and significant early signs of the incipient form. In the very early stages of phthisis percussion may be negative and misleading. Any abnormal inequality in the apicies should receive further investigation, although the note on the left side is normally less intense, more resonant and lower in pitch than that of the right.

Auscultation is by far the important procedure of physical examination of the chest. Unilateral weak or rough breathing with diminished voice conduction is as important as an increase in their intensity. Each patient should be made to cough followed by deep inspiration when sibilant, cracking or bubbling rales or the mucous click may be heard.

The important areas are the apex anterior and posterior inner lung borders anteriorly, axillary apex, interlobular fissure outlined by the scapular border when the arm is on the opposite shoulder. Prolonged expiration and cogwheel or interrupted breathing are often of significance.

Although early diagnosis may be made months before bacilli appear in the sputum, nevertheless diligent search should be made for confirmatory evidence of suspicion. A swab of mucous may be taken directly from the larynx or a series of examinations may be made of the early morning sputum. Spenglers digestion method or the method of centrifugation may succeed when ordinary methods fail.

The Roentgen rays are often valuable, especially as a confirmatory aid to diagnosis. The interpretation of the findings is an art that must be appreciated. There are few experts and the methods of physical diagnosis are entirely sufficient for most cases.

The tuberculin tests may be of great value in confirming our observations. Four varieties are popular with the profession. The ophthalmo tuberculin reaction, known as the Calmette test, may be used in the weaker and stronger solutions in alternate eyes. The danger of a severe reaction is now well appreciated in cases of phlyctenular conjunctivitis, or any suspicious tubercular manifestation in the eye or eyelids. The Morrow reaction obtained on the skin beneath the sternum may be of occasional value. The Von Pirquet with controls is often useful among children. Hypodermic tuberculin may be of value when other diagnostic methods have failed, and should be used with extreme caution.

When the diagnosis is assured I believe it is our duty and obligation to inform the patient in a kind and sympathetic manner as to the true nature of the disease. When this sub-conscious bond of truthfulness and personal interest is established the fighting qualities of the patient are greatly increased.

---

Bence-Jones Proteinuria.—Drs. T. R. Boggs and C. G. Guthrie Bull, *Johns Hopkins Hosp.*, December, 1912, describe a case associated with metastatic carcinoma. They present a table of 65 cases of myeloma and other extensive lesions of the bone marrow without the Bence-Jones reaction, and touch on various other interesting points.

